# End-to-End Retinex-Based Illumination Attention Low-Light Enhancement Network for Autonomous Driving at Night

**DOI:** 10.1155/2022/4942420

**Published:** 2022-08-20

**Authors:** Ruini Zhao, Yi Han, Jian Zhao

**Affiliations:** ^1^School of Automobile, Chang'an University, Xi'an, Shaanxi 710064, China; ^2^School of Automation Engineering, University of Electronic Science and Technology of China, Chengdu, Sichuan 611731, China

## Abstract

Low-light image enhancement is a preprocessing work for many recognition and tracking tasks for autonomous driving at night. It needs to handle various factors simultaneously including uneven lighting, low contrast, and artifacts. We propose a novel end-to-end Retinex-based illumination attention low-light enhancement network. Specifically, our proposed method adopts multibranch architecture to extract rich features for different depth levels. Meanwhile, we consider the features from different scales in built-in illumination attention module. We encode reflectance features and illumination features into latent space based on Retinex in each submodule, which could cater for highly ill-posed image decomposition tasks. It aims to enhance the desired illumination features under different receptive fields. Subsequently, we propose a memory gate mechanism to learn adaptively long-term and short-term memory. Their weight could control how many high-level and low-level features should be reserved. This method could improve the image quality from both different feature scales and feature levels. Comprehensive experiments on BDD10K and cityscapes datasets demonstrate that our proposed method outperforms various types of methods in terms of visual quality and quantitative metrics. We also show that our proposed method has certain antinoise capability and generalizes well without fine-tuning when dealing with unseen images. Meanwhile, our restoration performance is comparable to that of advanced computationally intensive models.^1^

## 1. Introduction

Object detection [1], classification [2], identification [3], tracking [4, 5], and semantic segmentation [6] have shown impressive results in intelligent traffic [7]. These technologies are mostly based on normal lighting and clear weather; most of them are not suitable for poor light conditions. Traffic safety statistics [8] point out that 51.1% of fatal car crashes in the US happen at night (from 6pm to 6am), especially under extremely low-light conditions. To improve the safety of intelligent driving, the fundamental goal is to resolve these degraded traffic scenes. Low-light image enhancement needs to deal with poor visibility, low contrast, and missing color information. It is a challenging task especially in countryside with extremely low light. It is necessary to keep the enhanced image visually natural while improving the image contrast and visibility and restoring the color information.

The earliest methods enhanced the brightness and contrast of low-light images based on histogram equalization. Subsequently, Retinex theory was proposed to decompose reflectance and illumination from low-light images. Classical histogram equalization and Retinex theory have developed many improvements in low-light image enhancement. Histogram equalization will merge certain gray levels, resulting in oversaturation and loss of regional information. Meanwhile, the noise of image maybe further amplified. The original Retinex low-light enhancement algorithm and subsequent improved algorithms considering its color distortion all need to obtain the illumination map first, but the priors used in the estimation of illumination map are all artificially produced. These methods show poor generalization. In recent years, data-driven neural networks for low-light enhancement methods have been widely proposed and improved. Most Retinex-based methods use two-stage strategy to decompose low-light enhancement process, and Retinex theory is generally used for preenhancement operation in the first stage. We incorporate the Retinex theory into the illumination attention module, and the whole network is trained in an end-to-end manner. The detailed methods are given in the related work.

The contributions of this paper are summarized as follows:

- We propose an end-to-end Retinex-based illumination attention low-light enhancement network. The proposed network incorporates illumination attention module based on Retinex and memory gate unit in classical convolutional neural network. It adopts multibranch architecture to extract rich features for different depth levels. Reflectance features and illumination features are encoded into latent space separately based on Retinex in each submodule. The built-in content learning branches are expected to preserve reflectance components and ensure image details. The built-in illumination attention mechanism aims to enhance the desired illumination features under different receptive fields.
- We propose a memory gate mechanism to reasonably use enhanced long-term memory and short-term memory. The gate architecture could adaptively learn the weight between different depth level features; it could control how many high-level and low-level features should be reserved. The outputs of these long-term and short-term memories are fused to recover the low-light images with realistic tone and normal illumination.
- Our method is compared with several state-of-the-art methods via comprehensive experiments. The results are measured in terms of visual quality and quantitative assessment. Moreover, to investigate the generalizability with our method, we employ the model trained on the BDD100K_test dataset to restore the test scenes in cityscape dataset. All results demonstrate the superiority of our method.

The rest of this paper is organized as follows.  Section 2 discusses related work.  Section 3 describes the proposed methods in detail, including image processing pipeline, network architecture, and loss function.  Section 4 shows experiment results. Finally, conclusions and future perspectives are drawn in Section 5.

## 2. Related Work

To effectively improve image visibility, enhance image contrast, and restore color information in low-light enhancement task, the recent methods can be divided into two categories, namely, physical model-based methods and learning-based methods.

### 2.1. Physical Model-Based Methods

#### 2.1.1. HE-Based Methods

Most physical model-based methods consider histogram equalization [9, 10] and their variations. Histogram equalization-based method changes the grayscale histogram of the original image from a certain scope to a uniform distribution. After being stretched, the distribution of the image becomes more uniform and reasonable, which enables enhancing the local contrast. However, this method usually cannot deal with brightness, texture details, and color synchronously, and there is still local exposure. Many improved histogram equalization-based methods have been proposed. Celik and Tjahjadi [11] proposed to use interpixel context information to enhance the contrast of input images, and the Frobenius norm is constructed using the correlation between image pixels and neighboring pixels to map the diagonal elements of the input histogram to the diagonal elements of the target histogram. Lee et al. [12] proposed a contrast enhancement algorithm (LDR) based on hierarchical difference representation of two-dimensional histograms by amplifying the grayscale differences between adjacent pixels. Reza [13] proposed the Contrast-Limited Adaptive Histogram Equalization (CLAHE) algorithm, which limits the enhancement of local contrast by setting a threshold and uses an interpolation algorithm to improve patches. The histogram equalization-based methods have achieved certain results, but the similar gray levels of the image will merge with each other, resulting in the loss of some gray levels and eventually the loss of image details. Some patches of the image may also be underexposed or overexposed.

#### 2.1.2. Retinex-Based Methods

Retinex theory is an important method proposed in 1963, which can adjust the brightness and improve the color. It decomposes the image into illumination components and reflectance component. It is characterized by compressed dynamic range and color constancy. Both single-scale and multiscale Retinex theories are classic methods. The scale factor in the single-scale Retinex (SSR) [14] will greatly affect the quality of image enhancement. Multiscale Retinex (MSR) [15] performs image enhancement by selecting different scale factors multiple times on the basis of SSR. However, these two methods may introduce noise, resulting in local color distortion. In response to this problem, a color recovery factor is introduced in MSRCR to adjust the proportional relationship of RGB channels in the original image, which can highlight the darker patches and eliminate color distortion. Hao et al. [16] proposed a novel Retinex-based low-light image enhancement method; the image decomposition is achieved in an efficient semidecoupled way. Specifically, the illumination component is gradually estimated only from the input image by the proposed Gaussian Total Variation model, while the reflectance component is jointly estimated by the input image and the intermediate illumination output. Hou et al. [17] proposed a new pixel-level nonlocal Haar transform-based illumination and reflectance decomposition method named NLHD, in which the illumination component is to reconstruct by the low-frequency coefficient of Haar transform on each similar pixel group, while the reflectance component is to realize by the rest of all high-frequency coefficients.

### 2.2. Learning-Based Methods

#### 2.2.1. CNN-Based Methods

DLN [18] consists of several Lightening Back Projection (LBP) blocks to perform the lightening and darkening process iteratively to learn the residual for normal-light estimation. It also proposes a Feature Aggregation (FA) block that adaptively fuses the results of different LBPs by making use of global and local features. Liang et al. [19] extracted feature guided by the illumination map and noise map to predict local affine coefficients in the bilateral space. Zhang et al. [20] adopt Maximum Entropy-based Retinex (ME-Retinex) model to construct the illumination enhancement network ICE-Net, and the network introduces a new constraint that the maximum channel of the reflectance image is consistent with the maximum channel of the light image. Zheng and Gupta [21] proposed a semantic-guided low-light enhancement network, designing an enhancement factor extraction network using depthwise separable convolution. This algorithm also considers high resolution by a recurrent image enhancement network and preserves semantic information by a semantic segmentation network. SurroundNet [22] is regarded as a novel extension of single-scale Retinex; it consists of several Adaptive Retinex Blocks (ARBlock). The ARBlock aims to estimate illumination by an adaptive surround function. TSDNet [23] proposed multiscale attention module to learn image distribution, and it also adopted two-branch extraction module and multifeature fusion module to integrate all characteristic information.

#### 2.2.2. GAN-Based Methods

Chen et al. [24] proposed a bidirectional generative adversarial network framework, adding global features to the network and using an adaptive weighting scheme for training, which can significantly improve the stability of training. WESPE [25] is also based on a bidirectional generator, which uses multiple residual blocks. Its main feature is the use of anticolor loss, antitexture loss, and smoothing loss to guide the update of networks. Yi et al. [26] use the UNet model as the attention generator and uses the global local discriminator structure to guide the generator to pay attention to both global and local information. LEUGAN [27] proposed an unsupervised generation network with attention guidance to handle the low-light enhancement task. It adapted an edge auxiliary module that restores sharper edges and an attention guidance module that recovers more realistic colors. UEGAN [28] embeds modulation and attention mechanisms to capture richer global and local features, combining fidelity loss and quality loss to preserve the desired characteristics. QAGAN [29] proposed a quality attention generative adversarial network embedded with a quality attention module. The proposed QAM allows the generator to effectively select semantic-related characteristics from the spatial-wise and adaptively incorporate style-related attributes from the channel-wise. Sun et al. [30] proposed a lightweight one-path conditional generative adversarial network, which consists of two complementary modules: the pixel-wise self-modulation (PSM) aims to adjust the feature distribution of the input image, and the channel-wise conditional modulation (CCM) aims to learn from the features of both the low-light and the reference image. PDGAN [31] adopts zeroDCE to recover illumination and use the residual dense block encoder-decoder structure to eliminate noise and adjust illumination. At the same time, the discriminator can integrate into fractional differential gradient masks to enhance details. RGNET [32] divides the enhancement task into two stages from coarse to precise, where it roughly amplifies the input image nonlinearly using an unsupervised network and builds a two-path network to restore image details, of which one is used for residual restoration and the other is used for contextual attention. Liu et al. [33] introduced fractional order differentiation into both generator and discriminator; it can better distinguish noise and high-frequency details. It also adopts a global discriminator to improve the overall reconstruction quality and restore brightness. Li and Chen [34] proposed to combine DCGAN (deep convolutional generative adversarial network) and MSRCP (multiscale Retinex with chromaticity preservation) algorithm to determine the mapping from night to day.

#### 2.2.3. Retinex-Based Learning Methods

Wei et al. [35] introduce Retinex theory into deep learning to achieve low-light enhancement tasks. Its overall idea has been continued in the subsequent low-light enhancement based on Retinex deep networks. Decomposition network aims to learn the different illumination components between paired low-light images and normal-light images. The reflectance component aims at denoising; the illumination enhancement network adopts an encoder-decoder architecture, and it also introduces a multiscale concatenation to adjust the illumination from hierarchical perspectives. Hu et al. [36] proposed a two-stage low-light enhancement method, including preenhancement and postrefinement. The RGB images are preprocessed and decomposed based on Retinex theory; then, the traditional image processing technology is applied to the illumination component, and just adaptive tone mapping is used to enhance the illumination component. The refinement network further improves the image quality by adversarial training to resist noise. Wu et al. [37] also construct Decom-RNet based on Retinex theory to decompose RGB images. Subsequently, multiple residual structures are used to adjust the illumination components, and deeper levels and the shallower level are mapped identically in Enhance-RNet. Finally, the refinement network is also used for noise removal. Weligampola et al. [38] proposed a novel deep learning pipeline, in which CNNs and GANs are optimized to minimize standard losses and adversarial loss. The proposed model divides the enhancement process into two parts; the decomp-net decomposes the images into reflectance and illumination based on Retinex, and it pays more attention to local information. The enhancement net focuses on local and global information. EUIEF [39] adopts a Luminous Transform (LT) to enhance underexposed images as a preprocessing module on the basis of their bright value. And the other enhanced image is generated by invert-Image enhancement method. The final enhanced image is the fusion between these two intermediate enhanced images. UFANet [40] adopts a feature attention network, combining pixel estimation and channel estimation to decompose low-light images into reflectance and illumination.

#### 2.2.4. Attention-Based Learning Methods

EnlightenGAN [41] integrates self-regularized attention in UNet generator to enhance underexposed regions and avoid overexposed regions. They adopt dual discriminator to direct global and local information, and they also leverage self-feature preserving loss to guide the training process and maintain the textures and structures. For attention mechanism, specifically, they reverse the normalized illumination channel of RGB image as self-regularized attention map and then multiply it with all intermediate feature maps to improve the visual quality consistently. Zhang et al. [42] employ mixed attention strategy to recover low-light image; specifically, they use nonlocal operation as spatial attention module to obtain a wider range of information in spatial domain; this way could guide the network to learn what should be in a seriously degraded scene. And they combine channel attention module to model the interdependence between channels for refining redundant color features. It is noted that their proposed module aims to deal with extreme dark raw images. Lv et al. [43] leverage two attention maps to guide the brightness enhancement and denoising tasks, respectively. The first attention map aims at concatenating on unexposed patches, and the second attention map focuses on identifying noises from real textures. They combine a reinforcement net to further enhance color and contrast. For attention mechanism, specifically, they use spatial attention in paired images to construct an inverted ue-attention map to correctly enhance the underexposed regions and avoid overenhancing normally exposed regions. For denoising task, they also leverage spatial attention to estimate noise map, avoiding unwanted blurring effect. Li et al. [44] propose a luminance-aware pyramid network to recovery low-light image in a coarse-to-fine way; they adopt three branches to extract feature and learn light mapping between input and target images. Besides, they use multiscale contrast feature block including channel split and shuffle and contrast attention mechanism. They leverage channel attention to focus on contrast feature and then to calibrate the weight of each channel based on the importance of candidate features. Wang et al. [45] adopt normalizing flow model to construct the one-to-many relationship in highly ill-posed tasks; they use an invertible network to regard the low-light images as the condition and then to map the distribution of normal-light image into a Gaussian distribution. The learned invertible network could deal with the other inference direction to restore low-light images. They propose to use the gradient maps in different direction as attention map for removing noise. Chen et al. [46] propose an attention-based broadly self-guided network; its architecture is a top-down self-guidance to efficiently incorporate multiscale and local features to recover images. The architecture consists of several multilevel guided dense blocks. They propose a global spatial attention module to generate better results; the module includes convolution, pooling, and spatial attention operation.

#### 2.2.5. Other Methods

*(1) Color Channel-Based Methods*. Zhao et al. [47] input partial channel combination to obtain multiple enhancement results. And a multiscale feature shuffle module (MFCS) aims to combine image features at different scales; this way makes the fusion images preserve more rich information. Atoum et al. [48] proposed a color-wise attention network (CWAN), in which CWAN_AB allows the color information drive attention, while CWAN_L focuses on enhancing image lightness and denoising simultaneously. It searches for any useful color cues in the low-light image to aid in the color enhancement process.

*(2) RNN-Based Methods.* Zhao et al. [49] adopted invertible neural networks (INN) for bidirectional feature learning to ensure the mutual propagation invertible; this invertible mechanism with bidirectional feature transformation could avoid color bias and recover the content for enhancement task. It also proposes a new recurrent residual attention module (RRAM) to gradually perform the desired color adjustments. Ren et al. [50] proposed a network that consists of two distinct streams to learn the global content and the salient structures of the clear image simultaneously in a unified network. It also adopts a novel spatially variant recurrent neural network as an edge stream to preserve edge details.

*(3) Superpixel Methods.* DALE [51] (dark region-aware low-light image enhancement) makes use of a visual attention module to accurately recognize dark regions and enhance brightness. This method adopts superpixel to estimate visual attention and can preserve the color, tone, and brightness of the original images and prevent normally illuminated areas of the image from being saturated and distorted.

## 3. Our Proposed Methodology

**Figure 1:**
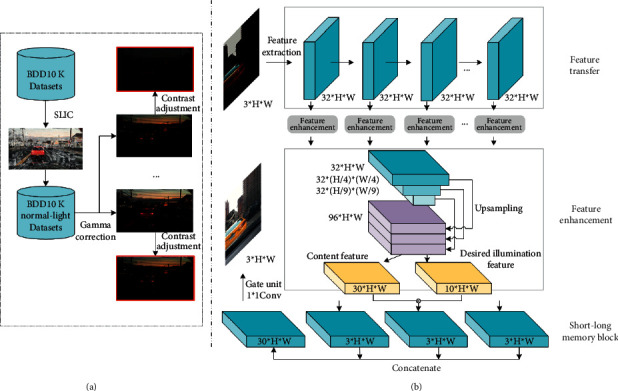
(a) Pipeline of constructing the proposed low-light simulation dataset. The details are shown in the next section. (b) The proposed network with three submodules. Feature transfer module includes ten convolution operations, the output of each feature transfer inputs both the next feature transfer submodule and feature enhancement submodule. Then, the feature pyramid is simultaneously used for content and illumination learning, and the desired illumination feature module is used to estimate the attention of illumination. Finally, short-term and long-term memory are all input into the memory gate unit; it adaptively learns different features by means of 1*∗*1 convolution operation to combine features from different channels.

In this section, we firstly introduce a data pipeline to generate paired low-light and normal-light images under extremely low light; these paired images are used for subsequent training of the proposed network. The image generation pipeline is shown in Figure 1(a). Next, we introduce the whole architecture of the proposed end-to-end illumination attention network for low-light enhancement task, shown in Figure 1(b); it mainly consists of three modules: (a) horizontal feature transfer module, (b) multiscale illumination attention based on Retinex module, and (c) long- and short-term memory fusion module. The entire architecture extends in both horizontal and vertical dimensions, and the features extracted from the original low-light images are transferred horizontally. Meanwhile, the features under different receptive fields are, respectively, sent to the feature enhancement module guided by the illumination attention. Finally, an adaptive gate mechanism is used to control the weights of long-term memory and short-term memory to obtain the final enhanced image.

### 3.1. Paired Image Generation Method

**Figure 2:**
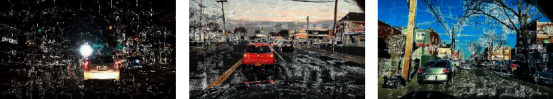
Example of superpixel image segmentation by means of SLIC.

**Figure 3:**
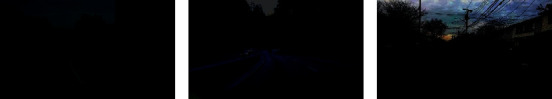
Generated extremely low-light images through the image processing pipeline.

We use the BDD10K dataset with normal daytime lighting as the ground truth, which is the most classic and largest open driving dataset. We use the classic superpixel segmentation algorithm and simple linear interactive clustering [52] (SLIC) to distinguish low-light and normal-light images. SLIC converts RGB images to CIELAB color space and five-dimensional feature vector. Then, we construct a distance metric for these feature vectors to cluster image pixels. The images in BDD10K are 720*∗*1280, we set the step size as 135, the estimated number of pixel blocks is 200, and the calculated number of pixel blocks is 180. To avoid underexposure, it is judged as a dark image patch when the average pixel value of each image patch is lower than 50, and when the number of dark image patches exceeds 45, we regard this image as a low-light image, and it will not be regarded as the ground truth for a low-normal-light image pair. As shown in Figure 2, they are examples of superpixel segmentation by means of SLIC. According to the above parameter settings, we get 1278 images in the BDD10K_test and 512 images in the BDD10K_val as normal-light images. The 1790 normal-lighting images are screened out and processed by the image processing pipeline. There are two steps: gamma correction and contrast adjustment. The output is defined as

(1) $$\begin{array}{c} I_{\text{light}}=\alpha \cdot \left(\text{scale}\cdot \text{gain}\cdot {\left(\frac{I}{\text{scale}}\right)}^{\gamma }\right), \end{array}$$

where *I* is the original input image, *I*_light_ is the processed image, the scale is a constant determined by the maximum pixel of input image, and the gain usually takes the default value 1. When *γ* > 1, the image pixels get darker under mapping relationship, and we choose *γ* ~ uniform(1,6). For contrast adjustment coefficient, we set *α* ~ uniform(0.1, 1). Several examples of the final generated low-light images are shown in Figure 3.

### 3.2. Network Architecture

The proposed end-to-end low-light enhanced network consists of three functional modules, and the whole architecture adopts a convolutional neural network as the baseline and incorporates an illumination attention module based on Retinex and memory gate unit. The input is low-light image and the output is an enhanced normal-light image. The multilevel feature transfer module includes 10 convolutional layers and keeps the feature map size consistent with the original image size all time. There is no downsampling and upsampling in the feature transfer flow at different depth levels. The output of each layer in the feature transfer module will be input to the Retinex-based illumination attention module, and the final gate unit mechanism will adaptively fuse the features under different receptive fields to obtain an enhanced image.

#### 3.2.1. Multilevel Feature Extract Module

CNN extracts feature through layer-by-layer abstraction, and different receptive fields could capture features at different levels. The low-level feature has a smaller receptive field and a strong ability to represent geometric detail information. Although the resolution is high, the ability to represent semantic information is weak. The receptive field of high-level features is large, and the semantic information representation ability is strong, but the resolution of the feature map is low, and the expression ability of geometric information is weak.

We propose a feature transfer flow structure, which contains a total of 10 convolutional layers. The input of the first layer is a three-channel image, and the outputs of each layer are all ℝ^*H*×*W*×32^. Not only do the features flow in the feature transfer stream, but they are also fed into the Retinex-based illumination attention feature enhanced module.

#### 3.2.2. Illumination Attention Feature Enhancement Module

Most previous Retinex-based low-light enhancement methods have been carefully designed with more constraints and parameters. The proposed illumination attention feature enhancement module simply adopts Retinex to decompose the input features into reflectance features and illumination features. According to Retinex theory, an image can be decomposed into an illumination map and a reflectance map; it is represented as

(2) $$\begin{array}{c} X=I\cdot R, \end{array}$$

where *X* is the RGB image and *I* and *R* are illumination and reflectance map, respectively.

As other Retinex-based methods, we regard reflectance component as content component. To take full advantage of the feature maps at different scales, we resize the feature maps at each depth level into three scales. Specifically, the features transferred from the feature extraction module are downsampled by means of average pooling to preserve the multiscale feature. The three-scale features are characterized by ℝ^*H*×*W*×32^, ℝ^(*H*/4)×(*W*/4)×32^, and ℝ^(*H*/9)×(*W*/9)×32^, respectively. Then, we concatenate these features and feed them into illumination learning submodule *G*_*I*_  and content learning submodule *G*_*c*_ based on Retinex. They are as follows:

(3) $$\begin{array}{c} X_{\text{cat}\_\text{feature}}=\text{conca}\left(X_{\text{fea}},{\text{Avgpool}}_{\text{kernel}=4}\left(X_{\text{fea}}\right),{\text{Avgpool}}_{\text{kernel}=9}\left(X_{\text{fea}}\right)\right),\\ X_{\text{inter}}=\sum_{i=0}^{9}G_{I}^{i}\left(X_{{\text{cat}}_{\text{fea}}}\right)\cdot G_{c}^{i}\left(X_{{\text{cat}}_{\text{fea}}}\right), \end{array}$$

where *X*_fea_ represents extracted features from different depth levels, Avgpool represents average pooling operation in PyTorch, kernel=4 means to average the pixels of four rows and four columns, and *conca* is a mapping function to concatenate features by channel. *G*_*I*_ tries to generate multiple intermediate illumination feature masks, *G*_*c*_ aims to produce multiple intermediate content feature masks, and *I* denotes i^th^ intermediate result; namely, we can get *i*^th^ intermediate content mask and *i*^th^ intermediate illumination mask.

The input of each attention module is 32-channel feature, and the outputs are illumination feature mask and content feature mask. Specifically, the input feature of illumination attentional module is ℝ^*H*×*W*×32^, for outputs, illumination mask *I* is ℝ^*H*×*W*×10^, and content mask *C* is ℝ^*H*×*W*×30^, where each channel of the illumination feature mask is copied to three channels to multiply the corresponding content feature mask. The illumination feature masks are expected to learn the illumination distribution of normal-light images guided by ground truth images. Then, we concatenate these intermediate results to get enhanced feature, and it is represented as

(4) $$\begin{array}{c} X_{\text{Enhan}}=\text{conca}\left(X_{\text{inter}}\right). \end{array}$$

#### 3.2.3. Memory Gate Unit

As mentioned above, we decompose features into content and illumination based on Retinex at different levels and scales. Then, we propose a memory gate unit that allows the network to adaptively control how many enhanced low-level features should be retained and how many enhanced high-level features should be stored. The formulation of the memory gate unit can be written as

(5) $$\begin{array}{c} \text{EnImages}=W_{\text{gate}}\left(X_{\text{Enhan}}\right), \end{array}$$

where *W*_gate_ denotes the function of 1*∗*1 convolutional layer, and it could learn the relationship between enhanced feature channels at different levels without loss of resolution.

### 3.3. Loss Function

To implement the low-light enhancement task, our loss function needs to simultaneously consider structure, content, and uneven lighting conditions. The optimization objective of the proposed network can be expressed as

(6) $$\begin{array}{c} L=\;L_{\text{str}}+L_{\text{content}}+L_{\text{region}}, \end{array}$$

where *L*_str_ , *L*_content_, and *L*_region_ are structural loss, content loss, and region loss, respectively, and their weighted coefficients all are 1 determined by multiple trials.

#### 3.3.1. Structure Loss

In addition to the low-light enhancement task, this paper also focuses on texture preserving and the brightness is allowed to be fluctuant around the ground truth. In particular, the captured low-light images usually show structural distortion during the enhancement process. The SSIM [53] between *x* and *y* is calculated as follows:

(7) $$\begin{array}{c} L_{ssim}=\frac{2\mu_{x}\mu_{y}+C_{1}}{\mu_{x}^{2}+\mu_{y}^{2}+C_{1}}\cdot \frac{2\sigma_{xy}+C_{2}}{\sigma_{x}^{2}+\sigma_{y}^{2}+C_{2}}, \end{array}$$

where *μ*_*x*_, *μ*_*y*_, *σ*_*x*_^2^, *σ*_*y*_^2^, and *σ*_*xy*_ are the mean of *x*, the mean of *y*, the variance of *x*, the variance of *y*, and the covariance of *x* and *y*, respectively. Both *C*_1_ and *C*_2_ are constants, *C*_1_=(*K*_1_ · *L*)^2^ and *C*_2_=(*K*_2_ · *L*)^2^, *K*_1_=0.01, *K*_2_  = 0.03, and *L*  = 255. *L*_*ssim*_ is in range (0, 1], in which 1 means that the two images are totally the same.

Meanwhile, SSIM performs well under a specific configuration, while MS-SSIM could maintain stable performance for images of different resolutions. Therefore, we combine SSIM and MS-SSIM for structured perception of images. It is noted that we use SSIM and MS-SSIM in the pytorch_msssim package. The loss function for MS-SSIM is defined as follows:

(8) $$\begin{array}{c} L_{ms-ssim}=\frac{1}{5}\prod_{i=1}^{5}{\text{weight}}_{i}\cdot \frac{2\mu_{xi}\mu_{yi}+C_{1}}{\mu_{xi}^{2}+\mu_{yi}^{2}+C_{1}}\cdot \frac{2\sigma_{xi,yi}^{i}+C_{2}}{\sigma_{xi}^{2}+\sigma_{yi}^{2}+C_{2}}. \end{array}$$

In the pytorch_msssim package, MS-SSIM downsamples the original input image four times in turn, *i* ∈ [1,2,3,4,5], *i*=1 represents the original size image, and the weights corresponding to each input scale are {0.0448, 0.2856, 0.3001, 0.2363, 0.1333}. The total structure loss is defined as follows:

(9) $$\begin{array}{c} L_{str}=L_{ssim}+L_{ms-ssim}. \end{array}$$

#### 3.3.2. Content Loss

Johnson et al. [54] proposed to adopt a pretrained VGG as a perceptual loss to model higher-level features images, which was widely used for many low-level vision tasks [55, 56]. We adopt a pretrained VGG to constrain the feature distance between enhanced images and corresponding ground truth, where (*x*_*i*_, *x*_*j*_) denotes the input low-light image, *G* represents low-light enhancement network, *GT* denotes the matched normal-light image, and *VGG* denotes the pretrained feature extract network. In this work, we use the output of the third block in VGG19 to extract higher-level features:

(10) $$\begin{array}{c} L_{\text{content}}={\left\|\left(VGG\left(G\left(x_{i},x_{j}\right)\right)-VGG\left(GT\right)\right)\right\|}_{1}. \end{array}$$

#### 3.3.3. Region Loss

For the uneven illumination in low-light enhancement tasks, we need to adaptively learn global illumination and local illumination to avoid overexposure or underexposure. We use the region loss proposed in MBLLEN [57] to separate the low-light areas from the image and then allocate a larger optimization weight to the first 40% of the darkest pixels in the entire image.

The region loss is defined as follows:

(11) $$\begin{array}{c} L_{\text{region}}=w_{L}\cdot \frac{1}{w\cdot h}\sum_{i=1}^{w}\sum_{j=1}^{h}\left({\|G}_{L}\left(x_{i},x_{j}\right),GT_{L}\|{}_{1}\right)+w_{H}\cdot \frac{1}{w\cdot h}\sum_{i=1}^{w}\sum_{j=1}^{h}\left({\|G}_{H}\left(x_{i},x_{j}\right),GT_{H}\|{}_{1}\right), \end{array}$$

where *w* *an*  *dh* are input images width and height, *G*_*L*_(*x*_*i*_, *x*_*j*_) and *GT*_*L*_ denote the low-light parts of the enhanced image and ground truth, *G*_*H*_(*x*_*i*_, *x*_*j*_) and *GT*_*H*_ are the rest parts of the images, *w*_*L*_ and *w*_*H*_ denote low-light regions and normal-light region weighted coefficient, and we use *w*_*L*_=4 and *w*_*H*_=1.

## 4. Experiments

### 4.1. Experiment Settings

We implemented our network by PyTorch on Tesla V100. We trained the network using Adam optimizer with default parameters for 200 iterations. The initial learning rate is 0.002. We adopt the strategy of learning rate exponential attenuation, and the attenuation coefficient is 0.99. For training, we used the proposed data pipeline to process normal-light images in BDD10 K_test dataset; then, we get 1278 paired extremely low-light and normal-light images. We compared our proposed network with ten state-of-the-art low-light enhancement methods, including based physical methods, based-learning methods, and attention-based learning methods, namely, LIME [58], MSRCR [14], RetinexNet [35], WESPE [25], DeepUPE [59], HDRNet [60], zeroDCE [61], EnlightenGAN [41], ABSGNet [46], and LLFlow [45].

### 4.2. Quantitative and Perceptual Comparisons

We quantitatively evaluated the ability of our network in low-light image enhancement. For fair comparison, we use the parameters provided by the recommended parameter settings for nonlearning methods. For learning-based methods, we retrain each method on the BDD10 K_test dataset. For testing, we compared the results of 720*∗*1280 resolution in BDD10K_val [62] and 512*∗*1024 resolution cityscapes datasets [63], and they are 512 and 1100, respectively. We leverage Peak Signal-to-Noise Ratio (PSNR), Multiscale Structural Similarity (MS-SSIM) [64], Learned Perceptual Image Patch Similarity (LPIPS) [65], Universal Quality Index (UQI) [66], and Feature Similarity Index Measure (FSIM) [67]. The higher PSNR indicates that our method could remove artifacts. For MS-SSIM, these results show our method could better preserve structural information. LPIPS as a perceptual metric aligned with human perception. UQI considers loss of correlation, luminance distortion, and contrast distortion to model image distortion. FSIM emphasizes that the human visual system understands the image mainly based on the low-level features of the image and replaces the statistical features in SSIM with image features. The quantitative comparison results of BDD10K_val (512 images) and cityscapes (1100 images) are as shown in the left and right parts of Table 1. The results we report are all average values.

We train the first group in the 720*∗*1280 resolution in our generated dataset; we can see that we achieve a dramatic improvement in most of quantitative evaluation metrics compared to other eight methods. For the parts of cityscapes dataset show, our method reveals the gratifying generalizability in most metrics. We can see that ABSGNet and LLFlow are relatively computationally intensive in Table 2; therefore, we train these two networks in 512*∗*512 due to memory constraints. For a fair comparison, we also train our methods at 512*∗*512 resolution. The last three rows of Table 1 compare the three sets of results. We could see that the quantitative results of these three methods are not much different, and each method has its own advantages. According to the results of the second group in Table 1, we can see that several attention-based low-light enhancement methods achieve better performance for most metrics on BDD10K_val and cityscapes datasets, which means attention-based methods are better able to focus on low-light regions and recover image texture and details.

On the premise of achieving better algorithm performance, we have to consider important factors that affect the deployment ability of the algorithm, including model parameters, multiply-accumulate operations, CPU inference time, and memory size. We compare three top-performing methods (ABSGNet, LLFlow, and ours) in Table 2. The test images we use are 240,360 and 720*∗*1280 RGB images, and we run them on Intel i7-8750H CPU with 16 GB RAM to compare CPU inference time, and all results are the average of five runs after the warm-up. It is evident that our method has the fewest parameters; it requires the least amount of computing resources. Our trained model memory is 22∼108X smaller. All training parameters of three methods will be used for inference, so the trained model size is consistent with model parameters. For FLOPs and CPU inference time, we infer on 240*∗*360 and 720*∗*1280 RGB images. Our method is about one-tenth of the other two for FLOPs. Our future work could improve the inference speed of the network by means of input parallelization.

**Figure 4:**
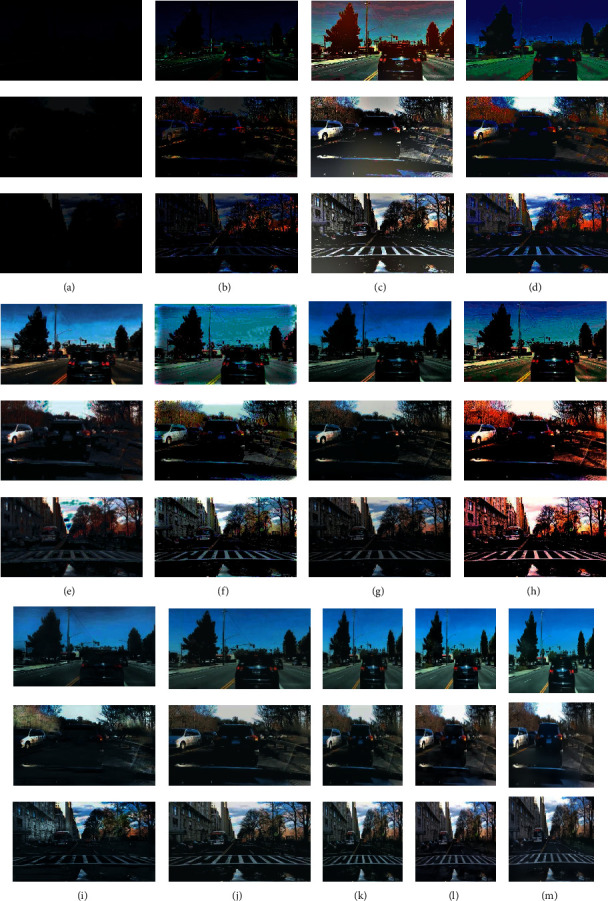
Examples of the enhanced BDD10 K_val dataset. The results of our method show the most realistic and natural lighting, meanwhile preserving the whole scene details. (a) Low-light image. (b) LIME. (c) MSRCR. (d) RetinexNet. (e) WESPE. (f) DeepUPE. (g) HDRNet. (h) zeroDCE. (i) Enlightengan. (j) Ours. (k) Ours 512*∗*512. (l) ABSGNet. (m) LLFlow.

**Figure 5:**
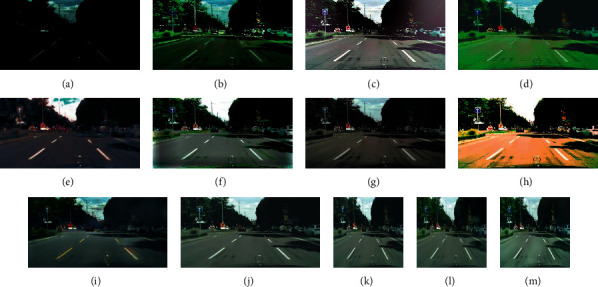
Visual comparison of Stuttgart city scene in cityscapes dataset. low-light images (a). LIME (b). MSRCR (c). RetinexNet (d). WESPE (e). DeepUPE (f). HDRNet (g). zeroDCE (h). Enlightengan (i). Ours (j). Ours 512*∗*512 (k). ABSGNet (l). LLFlow (m).

**Figure 6:**
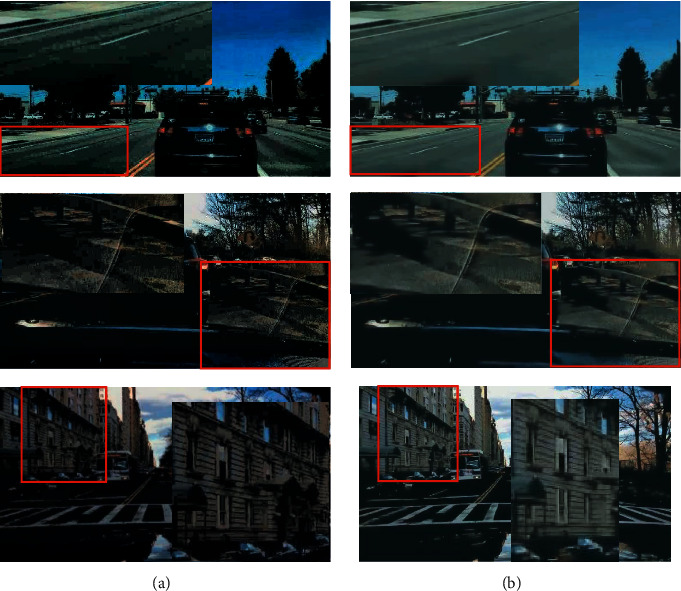
Intuitive performance of noise after low-light enhancement. HDRNet (a). Ours (b).

Figures 4 and 5 are visual comparisons of low-light enhancement methods on the BDD10K_val and cityscapes datasets, respectively.  Figure 4 shows the enhanced images of eleven methods for three low-light images. Comparing with Figure 4(b) to Figure 4(k), our method shows superior performances; we could preserve structured information, avoid color distortion, and eliminate artifacts simultaneously. We can see that the images enhanced by the lime and RetinexNet methods are oversaturated, resulting in unnatural color restoration. Meanwhile, their textures are not smooth. For MSRCR, WESPE, and DeepUPE methods, the entire generated images are extremely not smooth; they are with obvious artifacts and noise, and even large instances fail to maintain smooth edges. For zeroDCE, its brightness is not enhanced enough, and there is color oversaturation. For EnlightenGAN, its local enhancement effect is better, but the generation details need to be improved. Among the first eight compared methods, HDRNet could achieve similar visual results as our proposed method. Then, we zoom in the enhanced images of HDRNet and our method in Figure 6. Due to the memory limit and fair comparison, Figures 4(l)to 4(n) show the results of ABSGNet, LLFlow, and our method in 512∗512 size, and they all work well. Please refer to Table 1 for their quantitative evaluation.

We zoomed in the enhanced results for HDRNet and ours in Figure 5. The results show that our proposed method has good antinoise ability. Our network achieves highly ill-posed image decomposition based on multilevel and multiscale feature transfer. It could map illumination features well while preserving content features. In this way, the image details could be better maintained and the noise could be reduced. Compared with several methods for low-light enhancement, special noise removal modules are introduced [19, 31, 33, 43, 45]. Our network architecture used full-resolution features, not downsampling and upsampling operations to encode and decode features. In this way, it avoids the introduction of noise in the feature recovery process.

### 4.3. Generalizability

**Figure 7:**
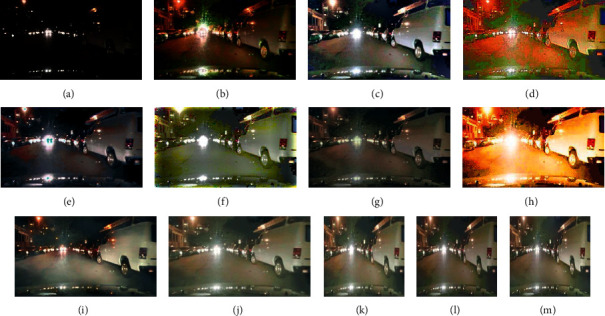
Enhanced real night scenes. The result of our method shows less artifacts compared with most of the methods, the lighting is consistent with realistic yellow hue, and there is no color distortion. Low-light images (a). LIME (b). MSRCR (c). RetinexNet (d). WESPE (e). DeepUPE (f). HDRNet (g). zeroDCE (h). Enlightengan (i). Ours (j). Ours 512*∗*512 (k). ABSGNet (l). LLFlow (m).

To improve the enhanced image quality, the existing state-of-the-art low-light enhancement methods build a model with high complexity, which makes the model easy to overfit on a specific dataset. To compare the generalizability of these models, we use the models trained on the BDD10K_test datasets to recover untrained Stuttgart city scenes in cityscapes datasets for quantitative and visual comparisons. The right part of Table 1 reports the average quantitative results over 1100 images. It can be seen that the generalizability of our proposed method performs better in most of metrics. Figure 5 is the visual comparison of the eleven methods, the images enhanced by LIME, RetinexNet, DeepUPE, and zeroDCE are too saturated, and there are also too much color distortion. For WESPE, the generated image is also far from smooth. HDRNet could not enhance brightness enough. For Enlightengan, there are some artifacts but its color recovery effect is better. Our method achieves some generalization advantage compared with these methods. But we can see that the enhancement results of ABSGNet and LLFlow show obvious advantages; they could not only enhance the brightness within the expectation but also restore the image color. Our method demonstrates superior performance on real night scenes from Figure 7. Our method shows the more realistic yellow tint along with ABSGNet and LLFlow.

## 5. Conclusion and Future Perspectives

Low-light image enhancement in traffic environment is a challenging task for intelligent driving tasks. We propose a data pipeline to generate paired low-light and normal-light images under extremely low-light, and these paired images are used for subsequent training of the proposed network. Our proposed method comprehensively considers detail preservation, color recovery, and illumination compensation for traffic scenes. We qualitatively and quantitatively compare our method with traditional and data-driven low-light enhancement methods, and the results show that our method has excellent performance in dealing with uneven illumination, low contrast, and artifacts. Meanwhile, there are fewer parameters, it is not easy to overfit, and the network shows better generalizability on the enhancement of other generated untrained low-light images and real low-light images. Our method is able to enhance low-light traffic scenes more effectively, increasing the overall brightness while making the final enhancement more natural, avoiding color distortion and numerous artifacts. Our work does better preliminary work for the subsequent target recognition and tracking of intelligent driving tasks, as well as semantic segmentation. The study shown in this paper can be extended along this direction. Our future work will also focus on the real-life deployment ability and further improving inference speed.

## Figures and Tables

**Table 1:** Quantitative comparison of the enhancement results.

	Methods	Metrics									
		BDD10K_val (512 images)					Cityscapes (1100 images)				
		msssim↑	PSNR↑	LPIPS↓	UQI↑	FSIM↑	msssim↑	PSNR↑	LPIPS↓	UQI↑	FSIM↑
First group (720*∗*1280)	LIME	0.650	9.717	0.163	0.313	0.949	0.623	12.073	0.179	0.205	0.950
	MSRCR	0.741	14.201	0.117	0.779	0.966	0.710	11.961	0.161	0.679	0.955
	RetinexNet	0.669	15.374	0.167	0.807	0.950	0.652	19.489	0.178	0.868	0.942
	WESPE	0.637	15.276	0.136	0.783	0.943	0.617	18.056	0.160	0.846	0.926
	DeepUPE	0.677	14.172	0.148	0.530	0.962	0.664	13.374	0.148	0.273	**0.958**
	HDRNet	0.798	16.129	0.079	0.727	0.970	0.774	16.194	0.105	0.629	0.953
	zeroDCE	0.657	13.264	0.161	0.506	0.965	0.639	12.753	0.158	0.236	0.955
	Enlightengan	0.711	15.998	0.113	0.812	0.951	0.638	17.548	0.154	0.801	0.931
	Ours	**0.842**	**21.863**	**0.062**	**0.915**	**0.971**	**0.800**	**21.974**	**0.097**	**0.897**	0.955

Second group (512*∗*512)	ours512∗512	0.859	22.247	0.075	0.923	**0.976**	**0.811**	21.992	0.114	0.899	**0.957**
	ABSGNet	0.848	**23.445**	**0.066**	**0.929**	0.973	0.773	**22.133**	0.105	**0.908**	0.947
	LLFlow	**0.860**	22.179	0.070	0.921	0.975	0.795	21.707	**0.112**	0.903	0.952

*Note.* The best result is in bold and the second best is underlined. Due to memory limitations and computing speed, we downsampled all generated images and ground truth to 240*∗*480 to calculate LPIPS and FSIM. The network used by LPIPS is Alex and the version is 0.0.

**Table 2:** Computation complexity and deployment ability metrics in 240*∗*360 (first group) and 720*∗*1280 (second group) RGB image.

	Methods	Trainable parameters↓	FLOPs (GMACs) ↓	CPU inference time (second)↓	Memory (Mb)↓
First group	ABSGNet	820*∗e*4	365.78	3.582	31.33
	LLFlow	3886*∗e*4	412.27	10.112	148.77
	Ours	**35 ** *∗ * ***e*4**	**30.62**	**1.119**	**1.37**

Second group	ABSGNet	—	3901.68	37.613	—
	LLFlow	—	4174.2	86.705	—
	Ours	—	**326.63**	**10.397**	—

## Data Availability

The data used to support the findings of this study are freely accessible. Please refer to the following links: https://doc.bdd100k.com/ and https://www.cityscapes-dataset.com/.
